# Age at first birth and risk of later-life cardiovascular disease: a systematic review of the literature, its limitation, and recommendations for future research

**DOI:** 10.1186/s12889-017-4519-x

**Published:** 2017-07-05

**Authors:** Nicole T. A. Rosendaal, Catherine M. Pirkle

**Affiliations:** Office of Public Health Studies, University of Hawaii, 1960 East-West Road, Biomed D104T, Honolulu, HI 96822-2319 USA

**Keywords:** Maternal age, Pregnancy in adolescence, Cardiovascular disease, Coronary heart disease, Stroke

## Abstract

**Background:**

Cardiovascular disease (CVD) is the principal contributor to the burden of disease and mortality worldwide. Previous studies observed associations between early age at first birth (AFB) and all-cause mortality. AFB may be associated with CVD both through physiological and sociobiological pathways. In this paper, we review the literature on AFB and CVD events and mortality. Additionally, we provide an overview of limitations of the current research and recommendations for future research.

**Methods:**

PubMed and Web of Science databases were searched for observational studies published between 1980-June 2016, investigating associations between AFB and CVD events and mortality. Data were extracted using a pre-defined list.

**Results:**

A total of 20 publications, reporting on 33 associations, were included in the review. Ten studies observed a positive association between early AFB and CVD while two studies observed a positive association between later AFB and CVD. Substantial methodological limitations were observed related to: operationalization of exposure categories, choice of reference category, sample size, follow-up time and possibly over adjustment.

**Conclusions:**

Early AFB is possibly related to CVD. More work, in particular from large cohort studies starting before reproductive age is reached, is needed to better investigate this relationship, and to ascertain causal pathways that may explain observed associations.

**Electronic supplementary material:**

The online version of this article (doi:10.1186/s12889-017-4519-x) contains supplementary material, which is available to authorized users.

## Background

Cardiovascular disease (CVD) is the principal contributor to the burden of disease and mortality worldwide [1]. Risk factors for CVD may differ between men and women. Until menopause, women appear at lower risk of CVD than men [2]. After menopause, risk of CVD increases in women and becomes similar to men [3]. Additionally, traditional CVD risk factors are less predictive of myocardial infarction in women compared to men [4]. These observations prompted extensive research on the associations of reproductive characteristics- parity, age at menarche, age at menopause- with CVD in later life [2, 5, 6]. Findings are inconclusive. In contrast, there is much less research investigating associations between maternal age at first birth (AFB) and CVD.

Early AFB possibly influences CVD events and mortality. Numerous studies observe associations between adolescent pregnancy and all-cause mortality [7–10]. Explanations range from the purely physiological to the social and behavioral. Having a family is a central feature of human existence and childbearing is a uniquely female experience. The age at which one gives birth, along with marital status and the circumstances of pregnancy, can have dramatic and enduring consequences on women and their families [11]. Adolescent pregnancy may contribute to cumulative adversity through a cascade of adverse events. Adolescent mothers frequently drop out of school and have lower economic opportunities [11–13]. Potential consequences of adolescent pregnancy such as low educational attainment, low income, social isolation, and violence have all been related to adverse cardiovascular health outcomes [14–17]. At the same time, adolescent pregnancy may trigger physiological changes in the body differently than adult pregnancy. For example, because they are still growing, weight-gain trajectories may differ for pregnant adolescents compared to adult pregnant women, with adolescents gaining and retaining more weight [18, 19]. Furthermore, adolescent pregnant women are exposed to physiological changes accompanying pregnancy that may irreversibly influence cardiovascular health earlier in life than women who have children later, thus increasing exposure durations [20]. In sum, AFB could influence cardiovascular health through multiple pathways.

This review has three objectives. First, we summarize the peer-reviewed literature examining associations between AFB and CVD (events and mortality). Second, we highlight important methodological limitations in the reviewed studies. Third, we give specific recommendations to improve the state of the literature.

## Methods

### Search strategy

We reviewed observational studies published in academic journals between 1980 and June 2016 that explicitly investigated associations between AFB and CVD events and/or mortality. To maximize the scope of the review, we included articles that considered total cardiovascular or circulatory disease, cerebrovascular disease, and coronary heart disease, but excluded peripheral arterial disease, rheumatic heart disease, congenital heart disease, deep vein thrombosis and pulmonary embolism. We conducted a title search in PubMed and Web of Science databases. For PubMed, we added medical subject heading (MeSH) terms. The full list of search terms is presented in Additional file 1.

### Study selection

Retrieved citations were imported into a literature review program, EPPI-reviewer 4 [21]. Titles and abstracts of retrieved citations were screened for eligibility after which, the full texts were retrieved and reviewed. Bibliographies of the selected articles were searched and a cited reference search was conducted to identify other eligible studies. Any study that reported a measure of association between AFB and CVD events or CVD mortality was considered eligible.

### Data extraction

Prior to data collection, we established a data-extraction form. This included: study design, location, year/duration of study, sample size, inclusion and exclusion criteria, population age, operationalization of exposure, point estimates, confounding variables and authors’ conclusion and interpretation of results. Concerning the authors’ interpretation of findings, we specifically extracted information on proposed causal pathways. NR extracted data for all retained articles. CP independently extracted data on a sample of five articles. No discernable differences in extracted data were noted between NR and CP and thus, the authors concluded there was no need for continued double data extraction. When information on sample size and/or number of cases was missing, the authors of the original article were contacted. Four authors were contacted for additional information and two replied.

## Results

### Study selection

**Fig. 1:**
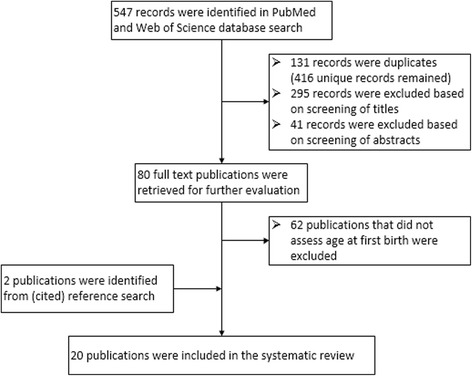
Study selection process for inclusion in systematic review

A total of 416 unique records were identified in the databases. Eighty full text papers that mentioned examining either AFB or parity in their abstract were retrieved for further evaluation. Eighteen studies were included [22–39]. All others did not provide a measure of association between AFB and CVD and were therefore excluded. Two publications [9, 10], were identified through (cited) reference search of the included articles, totaling the included publications to 20 (Fig. 1).

### Study characteristics

Six publications used a case-control design, while 14 employed prospective cohort designs. Characteristics of the case-control and cohort studies can be found in Tables 1 and 2 respectively. The six case-control studies all reported on a singular CVD outcome. Five of the cohort studies reported on only one CVD outcome, while nine reported on two or more CVD outcomes, bringing the total number of studied associations reported on to 33. Twenty-one of the 30 studied associations were on mortality, five were on non-fatal CVD events and seven were on a combination of fatal and non-fatal events. Eighteen studies examined AFB and two examined age at first pregnancy (AFP). From this point on, for the sake of brevity, we will use the term AFB unless we are specifically referring to AFP.

**Table 1:** Characteristics of Included Studies, by Year of Publication – Case-Control Studies (*N* = 6)

First author, publication year	Years of study	Location of study	Exclusion criteria	Cases	Controls	Age	Endpoint category^a^			Specific endpoint
							CHD	CV A	CVD	
Beard 1984 [23]	1960–1974	Rochester, Minnesota, USA	Unmarried women.	169, hospital admissions	338 age-matched (3y), seen at same clinic in same year as diagnosis for matched case, no prior CVD diagnosis	< 60	C			Incident cases of CHD (angina, myocardial infarction, sudden unexpected death)
La Vecchia 1987 [31]	1983–1986	Northern Italy	Women with recurrent infarction or uncertain diagnostic criteria.	202, hospital admissions	374, under age 55 admitted to same hospital with acute disease other than CVD, malignant, metabolic, hormonal or gynecologic (and not related to smoking/alcohol)	< 55 y, median age 47 (cases) 45 (controls: 24–54)	C			First time myocardial infarction
Talbott 1989 [37]	1973–1975	Pennsylvania, USA	Never married, no living relatives, prior history of CVD	67 cases (death records in one county)	73 neighborhood controls, alive, within 10 years age match, Caucasian.	25–64, mean age 54.6 (cases) 43.4 (controls)	M			Sudden cardiac death
Palmer 1992 [34]	1986–1990	Massachusetts, USA	Controls: people without phone-listing excluded (27%).	858, hospital admissions	858 controls from registry, 5 years age range, same precinct of residence.	45–69, mean age 60	E			First time, non-fatal myocardial infarction
Okamoto 2001 [33]	1992–1997	Nagoya, Japan	Aged ≥80 or <30	124 consecutive cases	248 age-matched (2 y) controls, one hospital (patients with gastro-intestinal disease) and one community control per case.	30–79, mean age 60 (cases) and 60.3 (controls)	C			First time subarachnoid hemorrhage
Bertuccio 2007 [24]	3 studies: 1983–1992 1988–1989 1995–2003	Italy	None mentioned	609, in hospital	1106 (in hospital for acute diseases unrelated to smoking and other recognized myocardial infarction risk factors)	18–79, median age 56 y (cases) 53 y (controls)	E			First time myocardial infarction

^a^This column provides an overview of study endpoint category (coronary heart disease (CHD), cerebrovascular disease (CVA), cardiovascular disease (CVD)) and whether it concerns events (E), mortality (M) or a combination (C) of events and mortality

**Table 2:** Characteristics of Included Studies, by Year of Publication – Cohort Studies (*N* = 14)

First author, publication year	Duration of study + years of FU	Location of study	Exclusion criteria	Cohort size	Number of cases (% of cohort)	Age at entry	Endpoint category ^a^			Specific endpoint
							CHD	CVA	CVD	
Colditz 1987 [26]	En: ‘76 F: until ‘82 FU: 6 y	USA	Diagnoses of CHD at entry, non-married. (Note: only registered nurses, 98% white)	119,963 women. 700,809 person years	308 (2.6%)	30–55 at entry	C			Incident cases non-fatal myocardial infarction or fatal CHD.
Cooper 1999 [27]	En: ‘34-‘39 F: until ‘90–‘91. FU: 51–57 y	Minne-sota, USA	Enrolled white college students. In FU excluded if missing information age CVD event, or missing CVD data in questionnaire	867. (714 self-administered FU, 153 proxy respondents (family members))	45 (35 non-fatal, 10 fatal) (5.2%)	Under 25 at entry, 63–81 at FU	C			Non-fatal and fatal CHD
Otterblad- Olausson 2004 [9]	En:‘85 census. FU: From ‘90-‘95. FU: 5 y	Sweden	Women who had first infant before the age of 30 between 1964 and 1989 are included. Exclusion: women who emigrated or died between census and follow up.	460,434 women	151 (total circulatory) (0.05%), no data reported for CHD and cerebrovascular.	Born ‘50 – ‘64 → 21 to 35 at census.	M	M	M	Total circulatory, CHD, cerebrovascular mortality.
Yang CY 2006 [38]	En: ‘78/‘87 F: until ‘03 FU: 16–25 y	Taiwan	Included women with a first and singleton childbirth between 1978 and 1987.	1,292,462 women, 27,402,995 person years	189 (0.01%)	?		M		Subarachnoid hemorrhage mortality
Henretta^b^ 2007 [30]	En: ‘31/‘41 F: ‘92/‘02^a^	USA	First two follow up years excluded. People who didn’t survive until follow up excluded.	4335 women	Heart disease: 13.2%, stroke: 8.9% (parous women)	Birth cohort ‘31-‘41. Age at FU: 51–61 through 61–71	E	E		Presence of disease in 1994: heart, stroke. Self-reported.
Sakauchi 2007 [36]	En:‘88/‘90 F: until ‘03. FU: 13–15 y	Japan	Previous history of cancer. Missing target question item.	63,600 women at baseline, 817,669 person years	CHD: 458 (0.7%) Cerebrovascular: 1151 (1.8%)	40 to 79 at entry.	M	M		CHD mortality, cerebrovascular mortality
Yang L 2009 [39]	En: ‘91-‘92 F: until ‘04. FU: avg. 12.9 y	Sweden	Prior CVD. Missing information on OC use or smoking. Baseline self-reported natural menopause or HRT use.	45,729	Stroke: 285 (0.6%); Ischemic: 193 (0.4%) Hemorrhagic: 72 (0.2%)	30–49		C		Ischemic and Hemorrhagic stroke.
Grundy 2010 [29]	En: ‘35/‘68 F:’ from ‘80 to ‘03. FU: 23 y	Norway	Analysis restricted to ages above 45 (largely completed childbearing for women). Men and women who died before this age were excluded	744,784 women (7.2 million person years) 785,317 men (7.36 million person years)	3605 women (0.5%) 12,640 men (1.6%)	Register data born ‘35-‘68. Mortality at 45–68			M	Death from circulatory diseases
Chang 2011 [25]	En: ‘85 FU: until ‘05 FU: 20 y	Kangwha, Korea	Ever on OC. Missing info on age menarche, MP, BMI, BP, AFB, gravidity or parity.	3257 women. 48,313 person years	478 (14.7%)	PostMP, 55 or older at entry	M	M	M	CVD mortality, CHD mortality, stroke mortality
Gallagher 2011 [28]	En: ‘89-‘91 F: until ‘00 FU: 10/12 y	China	None mentioned	267,400 women. 2,565,433 person years	CVD: 4349 (1.6%), Stroke: 2776 (0.9%), CHD: 494 (0.2%).	30–60 at entry	M	M		CHD, ischemic and hemorrhagic stroke mortality.
Webb 2011 [10]	En: ‘70 F: until ‘06 FU: max 37 y	England and Wales	Included women estimated to have reached age 13 during ‘70s-‘90s, sampled members of census ‘71, ‘81 and ‘91.	1,913,595 person years	133 (0.007%)	13–49	M			Heart disease mortality
Merritt 2015 [32]	En: ‘92/‘00 F: Until ‘10. FU: mean 12.9 y	10 western European countries	DM, myocardial infarction, angina, stroke, cancer. Report of a prevalent disease that could influence important confounders. Missing lifestyle questionnaire, vital status or date of death. Women reported never having menstruated or missing information on all reproductive variables.	322,972 women	Circulatory: 2404 (0.7%); Cerebrovascular: 808 (0.3%); CHD: 732 (0.2%)	25–70 at entry	M	M	M	Total circulatory, cerebrovascular, and CHD mortality
Barclay 2016 [22]	En: ‘32 - ‘60 F: ‘90 - ‘12 FU: 22 y	Sweden	This is a sibling study: ID for both parents needed, no multiple births, no only children, no childless individuals, no variance on either mortality or AFB.	12,635 women, 27,183 men	4503 women (35.6% 10,067 men (37.0%)	People enter cohort in ‘90 or after age 45. Ages 45–80 throughout follow-up.			M	Circulatory deaths
Parikh 2016 [35]	En: ‘91 F: until ‘10 FU: median 12 years	USA	Missing reproductive and CHD risk factor information, missing follow up, prevalent or unknown history of CHD.	72,982	4607(6.3%)	Mean age at start of study 63.2	E			Coronary Heart Disease events

*En* entry in to study, *F* follow-up until, *FU* follow up time in years, *OC* oral contraceptives, *MP* menopause, *BMI* Body mass index, *BP* blood pressure, *AFB* age at first birth;

^a^This column provides an overview of study endpoint category (coronary heart disease (CHD), cerebrovascular disease (CVA), cardiovascular disease (CVD)) and whether it concerns events (E), mortality (M) or a combination (C) of events and mortality

^b^The data used for this article are drawn from a cohort study. However, the data on stroke and heart disease are cross sectional on presence of heart disease in 1994

The most recent study dated from May 2016 and the earliest study dated from 1984. Eight studies were conducted in the USA, seven in Western-European countries and five in higher income settings of Asia. The sample size of the studies ranged from 140 to 1716 women in the case control studies and from 867 to 1,292,462 in the cohort studies. For the cohort studies, the minimum and maximum durations of follow-up were five years [9] and 57 years [27]. While some studies started following women at young ages or from the time of their first delivery and thus recorded AFB at baseline and/or prospectively [10, 27, 38], the rest of the studies began following women later in life and recorded AFB retrospectively.

### Study findings

#### Summarizing study results

As will be highlighted more in depth in latter sections of the results, the appointment of reference category, operalization of exposure categories, and adjustment for covariates is highly divergent between studies, rendering it difficult to draw straightforward conclusions. Therefore, we broadly synthesize the results per cardiovascular outcome, but encourage readers to refer to Tables 3 and 4 in which information on risk categorization, reference group, sample size and number of events by exposure category, point estimates, confidence intervals, and covariate choices are schematically displayed. In these tables we also highlight potential sources of biases related to these parameters.

**Table 3:** Age at First Birth and CVD – Case Control Studies (*N* = 6), by Year of Publication

Case control studies							
First author, publication year	Age at first birth	Exposure cases: N (%)	Exposure controls N (%)	Point estimate (+CI) ^a^ *Base model*	Point estimate (+CI) ^a^ *Final model*	Adjusted for or matched on. If underlined, also adjusted in base model.	Notes on study considerations and limitations.
Beard *1984* [23]	< 20	Data not provided	Data not provided		1.9 (0.7–5.6)	D: age; SE: - BM: HT, DM; HB: smoking; R: -	No data on number of individuals per exposure group. Likely number for <20 group is small.
	20–24				**1.8 (1.1–3.3)**		
	≥25/n.a. ^b^				1 (Ref)		
La Vecchia *1987* [31]	< 20	21 (10.4)	23 (6.1)		**2.31 (1.1–4.9)**	^c^ D: age; SE: - BM: - HB: - R: -	Nulliparous group includes young women who did not have children yet.
	20–24	71 (35.1)	117 (31.3)		1.47 (0.9–2.4)		
	≥ 25	75 (37.1)	111(29.7)		1.39 (0.8–2.4)		
	Nullipara	35 (17.3)	123 (32.9)		1 (ref)		
Talbott [37] *1989*	<20	14 (26.9)	7 (10.3)	**3.4 (1.1–9.9)**	2.5 (0.5–12.8)	D: age at demise (base model) SE: - BM: - HB: smoking (final model); R: -	No adjustment for age in final model, despite much younger controls. Small sample size. Caucasian-only controls; no race/ethnicity information for cases.
	≥ 20	38 (73.1)	61 (89.7)				
Palmer *1992* [34]	< 18	34 (4.5)	5 (0.7)	6.8	**5.5 (1.7–17)**	D: marital status (and age-matched); SE: education, education spouse, occupation; BM: drug-treated HT, elevated serum cholesterol, drug-treated DM, family history MI, BMI; HB: smoking, coffee, alcohol, physical activity; R: conjugated estrogen use, age menarche, parity, menopausal status	Extensive adjustment in final model
	18	33 (4.3)	31 (4.2)	1.2	1.1 (0.6–2.2)		
	19	65 (8.5)	39 (5.3)	1.6	1.6 (0.9–2.8)		
	20–24	376 (49.4)	366 (49.4)	1	1 (ref)		
	25–29	177 (23.3)	224 (30.2)	0.7	0.8 (0.6–1.1)		
	30–34	61 (8.0)	58 (7.8)	1.0	1.3 (0.8–2.2)		
	≥ 35	15 (2.0)	18 (2.4)	0.8	1 (0.4–2.9)		
	<20	132 (17.3)	75 (10.1)	CI not provided	**1.7 (1.1–2.6)**		
	≥ 20	629 (82.7)	666 (89.9)		1 (ref)		
Okamoto *2001* [33]	< 27	61 (49.0)	146 (58.8)		1 (ref)	D: age; SE: educational level; BM: hypertension; HB: smoking; R: -	Cut-off for youngest group <27
	≥ 27	63 (51.0)	102 (41.2)		1.45 (0.9–2.3)		
Bertuccio *2007* [24]	< 20	58 (11.4)	73 (8.6)	1 (ref)	1 (ref)	D: age, study; SE: education; BM: BMI, Cholesterol, DM, obesity, HLD, HT; HB: Smoking, coffee, alcohol; R: parity, MP, HRT, family history AMI	Extensive adjustment in final model
	20–24	207 (40.7)	342 (40.1)	0.72 (0.5–1.1)	0.90 (0.6–1.4)		
	25–29	172 (33.9)	319 (37.4)	**0.65 (0.4–0.97)**	0.92 (0.6–1.5)		
	≥ 30	71 (14.0)	118 (13.9)	0.70 (0.4–1.1)	0.94 (0.6–1.6)		

*D* demographics, *SE* Socio-economic, *BM* Biomedical, *HB* Health behavior, *R* Reproductive, *HT* hypertension, *DM* diabetes mellitus, *MI* myocardial infarction, *AMI* acute myocardial infraction, *BMI* body mass index, *HLD* hyperlipidemia, *MP* menopause, *HRT* hormone replacement therapy

^a^Risk estimates used: Relative risk: Beard, La Vecchia, Talbot, Palmer. Odds ratio: Okamoto, Bertuccio

^b^Analysis with 25+ and never pregnant separately showed the same risk for those two groups, which is why the authors combined them

^c^Authors indicate that a multivariate model yielded the same/similar results

The bold data indicate significant results

**Table 4:** Age at First Birth and CVD – Cohort Studies (*N* = 14), by Year of Publication

Cohort Studies							
First author, publication year	Age at first birth	No. in cohort	No. study outcomes N (%)	Point estimate (+CI) ^a^ *Base model*	Point estimate (+CI) ^a^ *Final model*	Adjusted for or matched on. If underlined, also adjusted in base model.	Notes on study considerations and limitations.
Colditz *1987* [26]	<19	6671 ^b^	3 (0.004)		1.3 (0.4–4.3)	^c^ D: age; SE: - BM: - HB: - R: -	Very few events in the exposure category of <19. Short follow-up in a relatively young age group.
	20–22	145,708	42 (0.029)		0.9 (0.6–1.2)		
	23–25	257,170	108 (0.042)		1 (ref)		
	26–29	151,206	82 (0.54)		1.1 (0.8–1.5)		
	≥ 30	77,091	35 (0.045)		0.8 (0.5–1.2)		
Cooper *1999* [27]	15–24	7996 ^b^	8 (0.100)		1.29 (0.5–3.0)	D: age; SE: - BM: - HB: - R: -	Number of outcomes very low in all exposure categories
	25–29	17,698	14 (0.079)		1 (ref)		
	30–32	5341	3 (0.056)		0.71 (0.2–2.5)		
	33–43	3715	8 (0.215)		**2.9 (1.2–6.9)**		
Otterblad- Olausson *2004 (CVD)* [9]	<20	60,686	41 (0.07)	**2.2 (1.5–3.1)**	**1.8 (1.2–2.6)**	D: age; SE: background SES in 1960 (parents), socio-economic position (‘90), family situation (‘90), welfare dependency (‘90) BM: - HB: - R: Parity	Conducted amongst women aged 30–45, follow-up time of 5 years, resulting in low number of events. Some women still having children.
	20–29	399,748	110 (0.03)	1 (ref)	1 (ref) ^d^		
Otterblad- Olausson *2004 (CHD)* [9]	<20	60,686	Data not	**2.8 (1.5–5.4)**	**2.2 (1.2–4.3)**		
	20–29	399,748	provided	1 (ref)	1 (ref) ^d^		
Otterblad- Olausson *2004 (CeVD)* [9]	<20	60,686	Data not	1.5 (0.8–2.9)	1.4 (0.7–2.7)		
	20–29	399,748	provided	1 (ref)	1 (ref) ^d^		
Yang CY *2006*	<26	859,942	102 (0.012)	1 (ref)		D: - SE: - BM: - HB: - R: Parity	No adjustment for participant age is reported (while due to study set-up women with older age at first birth were older at follow-up). Cut-off for youngest group <26
	26–30	372,895	70 (0.019)	**1.78 (1.3–2.4)**			
	≥ 31	59,625	17 (0.029)	**2.96 (1.8–5.0)**			
	Continuous			**1.10 (1.06–1.1)**	**1.08 (1.04–1.1)**		
Henretta *2007* *Heart disease* [30]	<20	991	Data not provided		**0.36 (P:<.01)**	D: age, race, US-born, unmarried at first birth, marital status; SE: Father’s education, education, log net worth, log income; BM: - HB: - R: Birth >39, birth interval, parity	No information on the number of outcomes per exposure group; no confidence intervals
	≥ 20	2956					
Henretta *2007* *Stroke* [30]	<20	991	Data not provided		−.03 *P*-value or CI not provided		
	≥ 20	2956					
Sakauchi *2007* CHD [36]	<23	Data not provided	121		1.09 (0.8–1.5)	D: age, study area; SE: - BM: - HB: - R: -	No denominator information provided
	23–25		116		1 (ref)		
	≥ 26		131		1.17 (0.9–1.5)		
Sakauchi *2007* (*CeVD*) [36]	<23	Data not provided	291		1.19 (0.99–1.4)		
	23–25		294		1 (ref)		
	≥ 26		326		1.07 (0.9–1.3)		
Yang L *2009, Ischemic stroke* [39]	<23	11,942	68 (0.57)	1.1 (0.7–1.5)	1.0 (0.6–1.5)	D: age; SE: education; BM: BMI, high blood pressure, DM; HB: alcohol, smoking, physical activity; R: -	Young age at first birth category not compared to lowest risk category. Few cases for hemorrhagic stroke.
	21–25	9905	49 (0.49)	1 (ref)	1 (ref)		
	≥ 26	17,444	48 (0.28)	**0.6 (0.4–0.9)**	0.7 (0.4–1.1)		
Yang L	<21	11,942	28 (0.23)	**2.0 (1–4)**	1.8 (0.8–4.1)		
*2009* *hemorrhagic stroke*	21–25	9905	11 (0.11)	1 (ref)	1 (ref)		
	≥ 26	17,444	19 (0.11)	1.1 (0.5–2.3)	1.2 (0.5–2.6)		
Grundy *2010* [29] *Women*	<20	862,007 ^b^	572 (0.07)	**1.47 (1.3–1.6)**	**1.22 (1.1–1.3)**	D: age, year, region residence, log population size, marital status; SE: level of education; BM: - HB: - R: parity	
	20–24	3,200,462	1567 (0.05)	1 (ref)	1 (ref)		
	25–29	1,670,417	574 (0.03)	**0.67 (0.6–0.7)**	**0.81 (0.7–0.9)**		
	≥ 30	646,983	224 (0.03)	**0.60 (0.5–0.7)**	**0.78 (0.7–0.9)**		
Grundy *2010* *Men* [29]	<23	1,164,183 ^b^	2190 (0.19)	**1.39 (1.3–1.5)**	**1.23 (1.2–1.3)**		
	23–28	3,238,174	4804 (0.15)	1 (ref)	1 (ref)		
	29–34	1,313,342	1772 (0.13)	**0.88 (0.8–0.9)**	**0.93 (0.9–0.99)**		
	≥ 35	473,472	571 (0.12)	**0.87 (0.8–0.95)**	0.93 (0.9–1.01)		
Chang *2011* *CVD* [25]	15–19	844	144 (17.1)	1.00 (ref)	1.00 (ref) ^d^	D: age at entry; SE: education, occupation; BM: BMI, HT HB: drinking, smoking R: - For CVD there was a mention of a fourth model in which the authors additionally corrected for reproductive variables and marital status, which did not change the findings.	Extensive adjustment in final model. Few CHD events.
	20–22	1646	230 (14.0)	**0.74 (0.6–0.9)**	**0.74 (0.6–0.9)**		
	≥ 23	787	104 (13.2)	**0.75 (0.6–0.96)**	**0.76 (0.6–0.98)**		
Chang *2011* *CHD* [25]	15–19	844	12 (1.42)	1.00 (ref)	1.00 (ref) ^d^		
	20–22	1646	24 (1.46)	0.88 (0.4–1.8)	0.89 (0.4–1.8)		
	≥ 23	787	11 (1.40)	0.93 (0.4–2.1)	0.90 (0.4–2.1)		
Chang *2011* *Stroke* [25]	15–19	844	87 (10.3)	1.00 (ref)	1.00 (ref) ^d^		
	20–22	1646	142 (8.6)	**0.76 (0.6–0.99)**	0.78 (0.6–1.02)		
	≥ 23	787	68 (8.6)	0.80 (0.6–1.1)	0.84 (0.6–1.2)		
Gallagher *2011* *CHD* [28]	<20	12,460	43 (0.35)		0.93 (0.7–1.3)	D: age; SE: - BM: - HB: - R: -	Young age at first birth category not compared to lowest risk category.
	20–24	72,570	239 (0.33)		1 (ref)		
	25–29	124,044	101 (0.08)		**0.75 (0.6–0.95)**		
	≥ 30	45,041	37 (0.08)		0.85 (0.6–1.2)		
Gallagher *2011* *Ischemic stroke* [28]	<20	12,460	75 (0.60)		1.23 (0.96–1.6)		
	20–24	72,570	309 (0.43)		1 (ref)		
	25–29	124,044	141 (0.11)		0.88 (0.7–1.1)		
	≥ 30	45,041	50 (0.11)		0.95 (0.7–1.3)		
Gallagher *2011* *Hemorrhagic* *Stroke* [28]	<20	12,460	178 (1.43)		1.09 (0.9–1.3)		
	20–24	72,570	850 (1.17)		1 (ref)		
	25–29	124,044	423 (0.34)		**0.84 (0.7–0.9)**		
	≥ 30	45,041	132 (0.29)		**0.81 (0.7–0.97)**		
Webb *2011* [10]	N/A	1,021,417 ^b^	46 (0.0045)		1 (ref)	D: age, decade; SE: - BM: - HB: - R: -	Few events in <20 category. Nulliparous group includes young women who did not have children yet.
	<20	159,716	16 (0.0100)		1.25 (0.7–2.2)		
	≥ 20	732,462	71 (0.0097)		0.93 (0.6–1.4)		
	<20	159,716	16 (0.0100)		1.35 (0.8–2.3)		
	≥ 20	732,462	71 (0.0097)		1 (ref)		
Merritt *2015* (FTP) *(CVD)* [32]	<21	39,201	304 (0.78)		1.15 (0.99–1.3)	D: age, site; SE: education; BM: BMI; HB: physical activity, smoking (duration and intensity); R: menopausal status.	Extensive adjustment in final model and no unadjusted model for comparison.
	21–23	71,322	497 (0.70)		1.11 (0.98–1.3)		
	24–25	52,056	359 (0.69)		1.05 (0.9–1.2)		
	26–30	75,927	557 (0.73)		1 (ref)		
	≥ 31	27,027	237 (0.88)		1.06 (0.9–1.2)		
Merritt *2015* (FTP) *(CeVD)* [32]	<21	39,201	97 (0.25)		1.14 (0.9–1.5)		
	21–23	71,322	150 (0.21)		0.96 (0.8–1.2)		
	24–25	52,056	122 (0.23)		1 (ref)		
	26–30	75,927	199 (0.26)		0.97 (0.8–1.2)		
	≥ 31	27,027	79 (0.29)		0.90 (0.7–1.2)		
Merritt *2015* (FTP) *(CHD)* [32]	<21	39,201	105 (0.27)		1.14 (0.9–1.5)		
	21–23	71,322	140 (0.20)		0.96 (0.7–1.2)		
	24–25	52,056	106 (0.20)		1 (ref)		
	26–30	75,927	166 (0.22)		0.99 (0.8–1.3)		
	≥ 31	27,027	74 (0.27)		1.10 (0.8–1.5)		
Barclay 2016 *Women* [22]	15–19	2998	1148 (38.3)	**1.18 (1.1–1.3)**	**1.25 (1.1–1.4)**	D: birth cohort [2]; SE: (sibling fixed effect model) age of person’s mother at time of their own birth, attained socioeconomic status, educational attainment, marital status; BM: - HB: - R: Completed parity	
	20–24	5232	1894 (36.2)	**1.11 (1.02–1.2)**	**1.23 (1.1–1.4)**		
	25–29	3052	1022 (33.5)	1 (ref)	1 (ref)		
	30–34	1010	322 (31.9)	0.94 (0.8–1.1)	0.89 (0.7–1.1)		
	35+	343	120 (35.0)	1.14 (0.9–1.4)	1.09 (0.8–1.5)		
Barclay 2016 *Men* [22]	15–19	1469	661 (45.0)	**1.45 (1.3–1.6)**	**1.37 (1.2–1.6)**		
	20–24	9380	3595 (38.3)	**1.11 (1.1–1.2)**	**1.10 (1.03–1.2)**		
	25–29	9533	3395 (35.6)	1 (ref)	1 (ref)		
	30–34	4590	1623 (35.4)	0.98 (0.9–1.04)	1.03 (0.9–1.1)		
	35+	2211	793 (35.9)	0.98 (0.9–1.1)	0.97 (0.9–1.1)		
Parikh 2016 [35]	Nullipara	10,462	Data not provided. Total cases: 4607		1.00 (0.9–1.1)	D: age at enrollment; SE: income, education, neighborhood SES variables; BM: history of high cholesterol requiring pills, hypertension, diabetes; HB: smoking; nr of still births, nr of miscarriages, breastfed for >1 month	Extensive adjustment in final model
	<20	8780		**1.65 (1.5–1.8)**	**1.27 (1.1–1.4)**		
	20–24	29,803		**1.25 (1.2–1.4)**	**1.14 (1.1–1.2)**		
	≥ 25	23,937		1 (ref)	1 (ref)		

*D* demographics, *SE* Socio-economic, *BM* Biomedical, *HB* Health behavior, *R* Reproductive, *AFB* age at first birth, *AFP* age at first pregnancy, *HT* hypertension, *DM* diabetes mellitus, *CHD* coronary heart disease, *CeVD* cerebrovascular disease, *SES* socio-economic status, *BMI* body mass index, *FTP* full term pregnancy

^a^Risk estimates used: Relative risk: Yang C, Yang L, Barclay. Rate ratio: Colditz, Cooper, Otterblad, Webb. Hazard ratio: Chang, Gallagher, Merritt, Sakauchi. Odds ratio: Grundy, Parikh. Log odds ratio: Henretta

^b^Person years rather than individuals in group. Percentage is based on N / person years

^c^Authors indicate that a multivariate model yielded the same/similar results

^d^For intermediate models, see original publications

The bold data indicate significant results

### Mortality

#### Cardiovascular disease (CVD)

In the five cohort studies reporting on CVD mortality, young AFB was positively associated with CVD mortality. In one of the five studies [32], the confidence interval crossed the one (HR: 1.15, 95%CI: 0.99–1.3) (Table 4).

### Coronary heart disease (CHD)

#### CHD overall

In four [9, 10, 25, 32] out of six studies reporting on CHD mortality, the youngest AFB group was at highest risk for CHD mortality. In three of these studies [10, 25, 32], the confidence interval crossed the 1 (Table 4). In the fifth study [28], women with an AFB of 25–29 had a lower HR (0.75, 95%CI: 0.6–0.95) compared to women with an AFB of 20–24 (reference category). Women in the youngest AFB group, <20, were not compared to women in the lowest risk group (AFB of 25–29) and the CI largely overlapped 1 when comparing those <20 to the reference group of AFB 20–24 (HR: 0.93, 95%CI: 0.7–1.3). In the sixth study [36], women with an AFB of ≥26 and women with an AFB of <23 were at higher risk compared to women with an AFB of 23–25, but this finding was not statistically significant (HR: 1.09, 95%CI: 0.8–1.5 and HR: 1.17, 95%CI: 0.9–1.5 respectively).

#### Sudden cardiac death

One case-control study [37] reporting on sudden cardiac death observed a positive association of young AFB with sudden cardiac death when adjusted for age. This association was attenuated when the authors adjusted for smoking, but no longer for age (Table 3).

### Cerebrovascular

#### Stroke overall

In all four cohort studies [9, 25, 32, 36] reporting on cerebrovascular death (stroke), the women with the youngest AFB were at greatest risk. All associations attenuated in the fully adjusted models with point estimates ranging from 1.14 to 1.4 and confidence intervals just crossing the 1 (Table 4).

#### Ischemic stroke, hemorrhagic stroke and subarchnoid haemorrhage seperately

One study [28] reported on mortality from ischemic stroke and hemorrhagic stroke. Women with an AFB <20 compared to women with an AFB of 20–24 had an HR of 1.23 (95%CI: 0.96–1.6) and 1.09 (95%CI: 0.9–1.3) for ischemic and hemorrhagic stroke, respectively.

One cohort study [38] reporting on subarachnoid hemorrhagic stroke observed an increased relative risk for women with a first birth at age 26–30, as well as for women aged older than 30 at first birth, compared to women <26 at first birth. In an additional model, in which AFB was analyzed as a continuous variable, the study observed an increased relative risk for subarachnoid hemorrhagic stroke for each year increase in age at first birth (Table 4).

### Events

#### Incident diagnosis of acute non-fatal myocardial infarction

All three studies [24, 31, 34] reporting on acute non-fatal myocardial infarction, observed women with an early AFB at highest risk (Table 3). It is important to note that the reference category is nulliparous women in the study by La Vecchia et al. [31] In the study by Bertuccio et al. [24] the association attenuated in their fully adjusted model.

#### Presence of heart disease

One study [30] observed a higher log odds ratio (0.36, *P* < 0.01) for the presence of heart disease in women with an AFB of <20 compared to 20 or older (Table 4).

#### Presence of stroke

One study [30] observed no evidence of an association of an early AFB and the presence of stroke (Table 4).

### Mixed outcomes (mortality and events)

Six studies, two case-control and four cohort studies examined a combination of fatal and non-fatal events. Four [23, 26, 27, 35] of the studies examining AFB and CHD events reported the young AFB group at higher risk compared to the reference group, but this was statistically significant in only one [35] (Tables 3 and 4). Cooper et al. [27] additionally observed a positive association between older AFB (33–43) and CHD. A case-control study by Okamoto et al. [33] reporting on fatal and non-fatal subarachnoid hemorrhage observed an increased risk with an AFB of 27 or older compared to <27 (OR: 1.45; 95%CI: 0.91–2.33).

Yang et al. [39] reported on fatal and non-fatal ischemic and hemorrhagic stroke separately. Women with an AFB ≥26 compared to 21–25 were at lower risk for ischemic stroke, but the association attenuated after adjusting for age, socioeconomic, biomedical and health behavior factors. For hemorrhagic stroke, women with an AFB of <21 had a relative risk of 2.0 (95%CI: 1.0–4.0) compared to women with an AFB of 21–25, but this finding attenuated when adjustment was made for a number of socioeconomic, biomedical and health behavior variables (relative risk:1.8; 95%CI: 0.8–4.1).

### Highlighting important methodological differences and limitations

#### Operationalization of exposure categories

The vast majority of studies investigate early AFB rather than late AFB. The age cut-offs differ from one study to another. The youngest AFB category was defined as follows: one study used <19; 12 studies used <20; two studies used <21; and one study each applied <23, <25, <27 as cut-offs. One study [34] used both <18 and <20 as an early AFB cut-off. Another study [33] used a cut-off of <26 and additionally, looked at AFB as a continuous variable. The oldest AFB cut-offs were ≥31 in two studies, ≥32 and ≥33 in one study each and, ≥35 in two studies, all other studies had a lower cut-off (Table 3). In general, authors did not justify their choice of cut-off.

#### Choice of reference category

The studies vary in the appointment of the reference group. A small number of studies selected the youngest or oldest age category as the reference category, while most studies selected one of the intermediate age groups as the referent group. A few studies include nulliparous women in their analyses and appoint that group as the reference group. Most studies limited their analyses to parous women.

#### Sample size, age at follow-up and follow-up time

There is large variation in study sample size, years of follow-up and age at follow-up (Tables 1 and 2). Studies with a small sample size, short follow-up, young age at follow-up, or a combination of the three are likely to record fewer CVD events. Four cohort studies [9, 10, 27, 39] report on one or more associations between AFB and a CVD event with fewer than 150 total events.

#### Confounding variables and proposed pathways

A large variety of potential confounding variables were included in the studies. Only half of the studies provided explanations for their covariate choices [9, 10, 22, 23, 25, 28–30, 35, 39]. Explanations included “established risk factors” [39], “potential confounding effects” [23] and “a priori defined plausible confounder” [28]. Three articles [22, 29, 30] explicitly presented a theoretical framework, while for the other studies a theoretical underpinning was implied. A number of studies present different statistical models in which they increasingly adjust for covariates [9, 22, 24, 25, 29, 35]. While intermediate variables are hypothesized, none of the articles applied methods such as structural equation modelling or mediation modelling to inform theories on possible pathways. In the fully adjusted models, all but one study adjusted for age. Eight studies adjusted for one or more other demographic covariates besides age. Eleven studies adjusted for one or more socio-economic covariates. Eight studies adjusted for one or more biomedical covariates. Nine studies adjusted for one or more health behaviors. Nine studies adjusted for one or more reproductive health covariates. Two studies [26, 31] with limited statistical adjustment mention that such adjustment for covariates did not alter their results, and thus presented only the unadjusted models.

Twelve studies [9, 22, 23, 25, 27–31, 34, 35, 38] observed an association between at least one of the exposure groups and the reference group. Five studies did not propose a pathway to explain the results, two studies proposed a physiological pathway and five studies considered a combination of physiological and social pathways.

## Discussion

### Summary of the literature examining associations between AFB and CVD

Results from the articles reviewed suggest an inverse association between AFB and CVD; women with early childbirths appear at heightened risk for CVD events and mortality. However, the results are not consistent and many associations attenuated notably after covariate adjustment. Ten studies observed a greater probability of CVD for women with an early AFB, while two studies observed a greater probability for women with later ages at first birth.

### Methodological limitations and their potential implications

The heterogeneity in the study findings very likely has to do with a number of methodological differences and limitations as documented in the results’ section. Here, we critically examine whether observed associations (or lack thereof) could be related to these methodological differences and limitations.

#### Operationalization of exposure categories

Almost none of the studies investigated the extreme ends of the distributions for reproductive ages. Only two studies [26, 34] examined adolescent childbirth (<19) and none of the studies looked at very young childbirth (<17). Yet, research on the association between AFB and all-cause mortality suggests the greatest mortality occurs among the youngest women [9]. Most studies used <20 as the youngest age category, while six used a higher cut-off (<21, <23, <25, <26 or <27).

Differences in the operationalization of the exposure groups likely contributed to the heterogeneity of the study results. Out of the six studies that used a higher cut-off (<21, <23, <25, <26 or <27) for their youngest age category, none reported an association of young AFB and CVD after covariate adjustment. If indeed the adolescent (<19) AFB-group is at highest risk for the studied outcome, combining that group with an older AFB-group, which is at lower risk, would lead to misclassification bias due to imperfect specificity and would bias the results towards the null-hypothesis. It is important to recognize that a number of studies reported on AFB as a secondary outcome and were not specifically designed to investigate the association between AFB and CVD. Furthermore, some studies indicate being unable to analyze early AFB due to a low number of individuals in that exposure category (college student cohort [27] and Nurses’ Health Study [26]).

#### Choice of reference category

The choice of reference category in several studies influences the interpretation of the reported findings. For some of the articles, it may have ‘masked’ an association with AFB. For example, Gallagher et al. [28] would have observed a lower HR for women with an AFB of 25–29 and possibly women with an AFB of ≥30, if they had defined AFB <20 as their reference group, rather than AFB 20–24. Also, a number of studies used nulliparous women as their reference group. For some studies, nulliparous women were combined with women who had not yet given birth [10, 31] or with an older AFB group [23]. For these, we cannot distinguish whether any observed associations are due to AFB, parity, or both. Thus, observed associations may be due entirely or partially to another factor (e.g. parity, infertility) and not the AFB category under examination. Moreover, when younger women who have not yet given birth are grouped together with nulliparous (older) women (who may have an underlying health condition related to their fertility status and CVD) [40], it may bias the findings.

#### Sample size, age of the cohort and follow-up time

Factors such as sample size, the age of the cohort, and follow-up time contributed to a low number of cases with disease in some studies, rendering it difficult to interpret reported findings. One study [27], for example, that observed a higher rate of CHD amongst women with an AFB of 33–43, compared to women with an AFB of 25–29, only recorded a total of 33 CHD cases in all parous women in the study. Another study [9], which followed women for 5 years, was conducted amongst relatively young women (aged 30–45 during follow-up). Despite the short follow-up and the young age of the women, the total number of CVD events among parous women in this study was 151, which was sufficient to reveal higher mortality for CVD amongst women with an AFB of <20 compared to an AFB of 20–29. However, the same study also examined the association between AFB and coronary heart disease and cerebrovascular events separately. Likely, the number of cerebrovascular events alone was too low to detect a difference between AFB categories. The authors do not report the number of cerebrovascular events.

The distribution of the populations studied in terms of reproductive exposure (AFB) may be restrictive. For example, the study by Colditz et al. [26] was conducted amongst registered nurses and as the authors highlight in the discussion, this sample may not be representative of US women in general, as only 1 % had an AFB of <20. This was likely the case for several other studies included in the review, such as Beard et al. [23] who only included married women.

Lastly, selective survival may have influenced findings in the studies that began following women later in life. Numerous studies have documented associations between early AFB and all-cause mortality [7–10]. Thus, associations may converge as a larger proportion of the highest risk women are selected out of the study sample.

#### Confounding variables and proposed pathways

A very wide range of covariates were included in many of the statistical models. Some models adjusted only for age, while others adjusted for several socioeconomic, biomedical, health behaviors and reproductive covariates. Few studies justified their choices for inclusion of covariates and few of the studies that did observe an association provided a thorough interpretation of their results. In the absence of more comprehensive conceptual frameworks, it is difficult to ascertain whether the statistical adjustment was sufficient, insufficient, or excessive. As Barclay et al. [22], Henretta [30], and Grundy and Kravdal [29] highlight in their studies, there might be different pathways by which reproductive exposure relates to (cardiovascular) disease. On the one hand, background variables such as childhood SES, as well as personality traits, may contribute to early childbearing (selection effect) [13]. At the same time, an early AFB could have consequences for SES by reducing future educational and occupational opportunities. It is difficult to discern to what extent early parenthood circumstances influence the relation between background SES and later life SES. As highlighted before, there is a similar uncertainty regarding physiological pathways. High childhood BMI is related to an early menarche [41], which in turn gives rise to more ‘opportunity’ for an early AFB (a selection effect). On the other hand, an early pregnancy may cause a higher BMI, since early pregnancy is related to greater weight gain during pregnancy compared to pregnancy in adulthood [18, 19]. And again in this relationship, childhood BMI is related to BMI in later life [41, 42]. It is therefore difficult to ascertain whether there is a need to statistically adjust for many of these covariates, as they may be intermediate variables.

Extensive statistical adjustment may have masked “real” associations between AFB and CVD. In a recent well-conducted study of Swedish women at age 40, younger age at first birth was associated with hypertension [43], which is one of the most important risk factors for CVD. When studies adjust for biomedical indicators such as BMI, hypertension, and cholesterol, which are markers of CVD, they implicitly assume an alternative pathway linking AFB to CVD that is not mediated by the classic risk factors. None of the authors who adjusted for such biomedical indicators provided a justification for an alternative pathway. They may have therefore over-adjusted their statistical models. Under adjustment may also be a concern. One study [38] that observed a higher risk for subarachnoid hemorrhage among women with an AFB of 26–30 and ≥31, compared to women with an AFB of ≤25, did not report adjusting for age. Since the women were tracked from the time of their first birth (between ‘78 and ‘87) until the end of follow-up in 2003, age at first birth was directly linked to participant age during follow-up. Women with a young age at first birth were younger compared to women with an older age at first birth in this sample and incidence rates of subarachnoid hemorrhage increase with age [44].

Overall, and acknowledging the limitations above, if we solely examine the results of the 14 studies that defined their young age at first birth group as <20 or younger, we observe the youngest AFB group at highest risk in 21 out of the 23 studied associations. In 13, the association was statically significant. As for the ten remaining associations from ten different studies, in three [24, 25, 37], the association was significant in the analyses that were age-adjusted only. In one study [28], an association would have likely been observed if the young age at first birth group had been compared to the age at first birth category with the lowest risk and, in five studies a low number of events [9, 10, 25] or a low number of women in the young age at first birth category [23, 26] likely limited study power.

### Study strengths and limitations

This is the first study to systematically review the literature on AFB and CVD events and mortality. Although we recognize the importance of aggregating results through meta-analysis, the combination of substantial caveats, such as the limited number of studies per CVD endpoint, differences in the operationalization of exposure, covariate choices, follow-up times and age at follow-up, rendered such an analysis inappropriate. Additionally, only English language articles were reviewed, potentially overlooking valuable publications in other languages.

**Table:** 

**Recommendations to improve the state of the literature**
- Investigate the distributional tails of AFB, especially very early and very late age at first birth. - Justify AFB exposure categorization. How were categories selected (theory, biological evidence, statistical considerations, etc.)? - Reference category selection should reflect underlying hypotheses about exposed and unexposed groups. - Assure sufficient sample size and follow up time, taking into account cohort-age (fewer events should be expected for younger cohorts). For smaller studies, a priori power calculations are advisable. - Improved use of theory to direct covariate choice and modelling approaches. Where appropriate, future research would benefit from more sophisticated use of tools for investigating pathways (e.g. directed acyclic graphics, mediation modelling, structural equation modelling, etc.) - Ideally, prospective studies that start well before reproductive age could help answer questions about selection effects (for example, are observed associations between early AFB and CVD linked to some common earlier life event such as obesity in childhood or a “risk-taking” personality)

## Conclusion

While not all findings are consistent, this review provides evidence supporting an association between AFB and CVD, which warrants further investigation. The study findings are relatively consistent when young AFB is defined as <20. The findings in this systematic review highlight the need for improvement in the methods when researching this topic. A well thought-out conceptual framework geared towards researching AFB specifically, is vitally important for future work. Large cohort studies that use a long follow-up duration and start well before reproductive age, as well as employ more advanced analysis techniques designed for investigating causal pathways, may prove useful to better understanding this important topic.

## Additional file

Additional file 1: Search Strategy. (DOCX 11 kb)

## Abbreviations

AFB: Age at first birth
AFP: Age at first pregnancy
CHD: Coronary heart disease
CVD: Cardiovascular disease
HR: Hazard ratio
OR: Odds ratio
