# Supplementary material for: Age at first birth and risk of later-life cardiovascular disease: a systematic review of the literature, its limitation, and recommendations for future research
Source: BMC Public Health. 2017 Jul 5;17:627. doi: 10.1186/s12889-017-4519-x (PMC5498883; doi:10.1186/s12889-017-4519-x)
Supplement: Additional file 1: — Search Strategy. (DOCX 11 kb) [file 12889_2017_4519_MOESM1_ESM.docx]

# ADDITIONAL FILE 1

**Search Strategy**

**PubMed:**

("maternal age"[MeSH Major Topic] OR parity[MeSH Major Topic] OR gravidity[MeSH Major Topic] OR "paternal age"[MeSH Major Topic] OR parity[Title] OR nullipar*[Title] OR primipar*[Title] OR multipar*[Title] OR "number of pregnancies"[Title] OR "number of births"[Title] OR gravidity[Title] OR "number of children"[Title] OR nonparous[Title] OR "age at first birth"[Title] OR "paternal age"[Title] OR "maternal age"[Title] OR "age at first pregnancy"[Title] OR "age at first childbirth"[Title] OR "teenage pregnancy[Title]" OR "adolescent pregnancy"[Title] OR "pregnancy history"[Title] OR "reproductive history"[Title] OR "age at birth"[Title] OR "fertility history"[Title] OR "reproductive factors"[Title] OR "early childbearing"[Title] OR "late childbearing"[Title] OR "reproductive risk factors"[Title]) AND ("cardiovascular diseases"[MeSH Major Topic] OR cardiovascular[Title] OR stroke[Title] OR strokes[Title] OR heart[Title] OR CHD[Title] OR CVD[Title] OR "cardiac disease"[Title] OR "coronary"[Title] OR mortality[Title] OR atherosclerosis[Title] OR "subarchnoid hemorrhage"[Title] OR "subarchnoid haemorrhage"[Title] OR "cardiac death"[Title] OR "myocardial infarction"[Title] OR "premature death"[Title] OR "intracerebral"[Title]) AND ("1980"[Date - Publication] : "3000"[Date - Publication]) NOT congenital[Title] NOT fetal[Title] NOT "during pregnancy"[Title] NOT congenital[Title] NOT fetal[Title] NOT "during pregnancy"[Title] NOT gestational[Title] NOT "in pregnant women"[Title] NOT preeclampsia[Title] NOT cows[Title] NOT rats[Title] NOT sheep[Title] NOT "in children"[Title] NOT infant*[Title] NOT "pregnancy induced"[Title] NOT offspring[Title] NOT newborn[Title] NOT "childhood diabetes"[Title] NOT "child mortality"[Title] NOT "multiparametric"[Title] NOT "multiparameter"[Title]

**Web of Science:**

TI=(parity OR nullipar* OR primipar* OR multipar* OR "number of pregnancies" OR "number of births" OR gravidity OR "number of children" OR nonporous OR "age at first birth" OR "paternal age" OR "maternal age" OR "age at first pregnancy" OR "age at first childbirth" OR "teenage pregnancy" OR "adolescent pregnancy" OR "pregnancy history" OR "reproductive history” OR "age at birth" OR "fertility history" OR "reproductive factors" OR "early childbearing" OR "late childbearing" OR "reproductive risk factors") AND TI=(cardiovascular OR stroke OR strokes OR heart OR CHD OR CVD OR "cardiac disease" OR"coronary" OR mortality OR atherosclerosis OR "subarchnoid hemorrhage" OR "subarchnoid haemorrhage" OR "cardiac death" OR "myocardial infarction" OR "premature death" OR "intracerebral") NOT TI=congenital NOT TI=fetal NOT TI="during pregnancy" NOT TI=congenital NOT TI="during pregnancy" NOT TI=gestational NOT TI="in pregnant women" NOT TI=preeclampsia NOT TI=cows NOT TI=rats NOT TI=sheep NOT TI="in children" NOT TI=infant* NOT TI="pregnancy induced" NOT TI=offspring NOT TI=newborn NOT TI="childhood diabetes" NOT TI="child mortality" NOT TI="multiparametric" NOT TI="multi parameter”
